# Supplementary material for: HSP110 Regulates the Assembly of the SWI/SNF Complex
Source: Cells. 2025 Jun 5;14(11):849. doi: 10.3390/cells14110849 (PMC12155415; doi:10.3390/cells14110849)
Supplement: Supplementary file 1 [file cells-14-00849-s001.zip › Pointeau et al. Supplementary table 1.pdf]

Supplementary table 1: Antibodies used for the western blot analysis.

Proteins	References	Species	Dilutions	Incubation times
ATR	Cell signaling #CST2790	Rabbit	1/1 000	Overnight, 4°C
Flag	Abcam #Ab125243	Mouse	1/1 000	Overnight, 4°C
Histone H3	Cell signaling #CST14269	Mouse	1/1 000	Overnight, 4°C
HSP110	Abcam #Ab108625<br>GeneTex #GTX50045	Rabbit<br>Mouse	1/1 000	Overnight, 4°C
Lamin B1	Santa Cruz #SG20682	Rabbit	1/1 000	Overnight, 4°C
pATR	Abcam #227851	Rabbit	1/1 000	Overnight, 4°C
SMARCA4	Cell signaling #CST52251	Mouse	1/1 000	Overnight, 4°C
SMARCC2	Cell signaling #CST12760	Rabbit	1/1 000	Overnight, 4°C
GAPDH	Santa Cruz #SC47724	Mouse	1/1 000	Overnight, 4°C
TBP	Cell signaling #CST44059	Rabbit	1/1 000	Overnight, 4°C
β-actin-HRP	Sigma Aldrich #A3854	Mouse	1/5 000	Overnight, 4°C
Anti-rabbit-HRP	Cell signaling #CST7074	Goat	1/10 000	1h, RT
Anti-mouse-HRP	Cell signaling #CST7076	Horse	1/10 000	1h, RT
